# Bioenergy and Breast Cancer: A Report on Tumor Growth and Metastasis

**DOI:** 10.1155/2016/2503267

**Published:** 2016-09-05

**Authors:** Alice Running, Mark Greenwood, Laura Hildreth, Jade Schmidt

**Affiliations:** ^1^College of Nursing, Montana State University, Bozeman, MT, USA; ^2^Department of Mathematical Sciences, Montana State University, Bozeman, MT, USA

## Abstract

As many as 80% of the 296,000 women and 2,240 men diagnosed with breast cancer in the United States will seek out complementary and alternative medicine (CAM) treatments. One such therapy is Healing Touch (HT), recognized by the National Center for Complementary and Integrative Health (NCCIH) as a treatment modality. Using a multiple experimental groups design, fifty-six six- to eight-week-old Balb/c mice were injected with 4T1 breast cancer tumor cells and randomly divided into intervention and positive control groups. Five days after tumor cell injection, mice in the intervention groups received HT either daily or every other day for 10 minutes by one HT practitioner. At 15 days after tumor cell injection, tumor size was measured, and metastasis was evaluated by a medical pathologist after necropsy. Tumor size did not differ significantly among the groups (*F*(3,52) = 0.75, *p* value = 0.53). The presence of metastasis did not differ across groups (chi-square(3) = 3.902, *p* = 0.272) or when compared within an organ (liver: chi-square(3) = 2.507, *p* = 0.474; lungs: chi-square(3) = 3.804, *p* = 0.283; spleen: chi-square(3) = 0.595, *p* = 0.898). However, these results did indicate a moderate, though insignificant, positive impact of HT and highlight the need for continued research into dose, length of treatment, and measurable outcomes (tumor size, metastasis) to provide evidence to suggest application for nursing care.

## 1. Introduction

In the United States, in 2013, it is estimated that more than 296,000 women and 2,240 men will be diagnosed with breast cancer [1]. The chance of a woman being diagnosed with breast cancer during her lifetime has increased from about 1 in 11 in 1975 to 1 in 8 today [2]. As with breast cancer in women, breast cancer in men has increased 27% over the past 25 years [3]. Breast cancer in men, while rare, most often occurs after the age of 60 and is often discovered at a late stage [4].

Histologically, carcinomas from both the male and female breasts are indistinguishable [5]. Men with breast cancer generally follow a protocol similar to that of female breast cancer patients, including a combination of surgery, radiation, chemotherapy, and antihormone therapies [6], all of which carry considerable risk and extremely unpleasant side effects [7]. Five-year survival rates for breast cancer for women range from 15% to 93%, with better prognosis accompanying early detection. In general the prognosis for male and female patients with breast cancer is similar, but overall survival rates are lower for men. This may be due to an older age at diagnosis and more advanced stage of disease when diagnosed. However, when survival is adjusted for age at diagnosis and stage of disease, outcomes for men and women are comparable [3]. Most breast cancer deaths are due to the spread of the disease to other parts of the body and its consequence on impairing the function of vital organs like lung, liver, and brain. These metastatic processes begin with the transformation of primary tumor cells into a “phenotype that promotes unregulated growth, angiogenesis, breakdown of the extracellular matrix, extravasation, entry of metastatic cells into the circulation, cell adhesion to the endothelium of target organs, extravasation, and subsequent growth in new organs” (p 2) [8]. The rate of metastatic breast cancer at initial diagnosis in the United States has not changed since 1975 for women under 50 years of age; however, because of widespread introduction and utilization of mammographic screening and increases in women using hormone therapy, increases have been noted in women over 50 years of age [9].

Increasingly, breast cancer patients are seeking complementary and alternative medical (CAM) treatment options [10]. Common alternative treatments include chiropractic or osteopathy, herbal medicine, massage, deep breathing, meditation, acupuncture, dietary changes, and yoga [11]. Some patients seek CAM treatment because of a belief in natural or holistic options, others seek it to deal with the unpleasant side effects of traditional allopathic medical treatment, and still others seek it because they are committed to trying any and everything that might help. More recently results from a survey of oncology outpatients found that 61.3% of the participants reported using CAM modalities following cancer diagnosis. Factors associated with CAM use were female gender and breast cancer diagnosis [12, 13]. In particular, those participants who used energy healing (bioenergy) reported significantly more benefit than nonusers.

Energy healing (or biofield therapy) is a broad term that encompasses several therapeutic techniques with diverse philosophical and geographical origins. What many of these traditions share is a belief that all living things possess a* bioenergy*, the balance or imbalance of which is related to health and illness. Energy healers in many traditions seek to balance the body's energy by conducting a flow of their own energy to the person seeking healing, thus increasing the body's capacity for self-repair. This can be done through either a hands-on or a hands-off or a no-contact approach (where the healer's hands are positioned above the body of the patient). Levin [14] suggests a taxonomy for classifying energy healing systems. The East Asian tradition (including schools such as Reiki and Qigong) is based on principles of traditional Chinese medicine, particularly the existence of* qi* or life force that flows through specified meridians or channels. The bioenergy tradition has its roots in Eastern Europe and is marked by a belief in the interconnectedness of spiritual and physical aspects of human life. The contemporary metaphysical tradition includes an eclectic mix of techniques allied with either the new age movement or some formal (often Christian) religion. Finally, the Western professional tradition includes such techniques as Therapeutic Touch and Healing Touch and is practiced extensively by those in the nursing profession.

Practitioners of Healing Touch (HT) propose that every person is surrounded by a subtle energy field and that disruption of this field results in illness or disease [15]. The HT practitioner, through compassionate intention during the transfer of healing energy from healer to patient, seeks to manipulate the energy field to restore health and promote the body's ability to heal itself. Positive preliminary results have been reported for the use of HT in the treatment of pain, cardiovascular complaints, behavioral symptoms related to Alzheimer's disease and other dementia, depression, and posttraumatic stress disorder diseases, and symptoms related to cancer and cancer treatment [16]. Wilkinson et al. [17] found increases in salivary secretory immunoglobulin A (a measure of immunocompetency) and decreases in stress and self-reported pain following HT treatment. Mitchell et al. [18] found that HT was “likely” to be effective for reducing fatigue in patients receiving high-dose chemotherapy.

Jain and Mills [19] reviewed 66 studies using several biofield therapies in the treatment of a range of medical problems including pain, dementia, cardiovascular problems, and cancer. Interventions included Healing Touch, Therapeutic Touch, Qigong, and Reiki. The authors concluded that there is moderate to strong evidence of the efficacy of these therapies in reducing pain in hospitalized, pain, and cancer patients, as well as moderate evidence of efficacy in improving dementia and anxiety symptoms. Jain and Mills call for better-controlled studies to further validate these preliminary results. In a recent literature review aimed at critically evaluating clinical trials in adult patients with cancer, Therapeutic Touch was found to have positive effects on pain, nausea, anxiety and fatigue, and life quality in addition to biochemical parameters [20].

There is a dearth of high-quality randomized controlled clinical trials investigating the efficacy of HT [21] and even fewer specifically assessing the utility of this therapeutic approach in the treatment of cancer. Cook and Guerreria [22] investigated the effects of HT on health related quality of life (HRQoL) in women undergoing radiation therapy for gynecological or breast cancer. HT resulted in better functioning in the vitality, pain, and physical functioning domains of the HRQoL measure when compared with mock treatment. Minimal decrease in natural killer cell cytotoxicity has been found in cervical cancer chemoradiation patients when compared to the marked decrease in those receiving relaxation training or usual care [23]. This same study also found significant improvement in depressed mood among those participants receiving the HT intervention. Judson et al. [24] found a trend for improvement in immunological profiles among ovarian cancer patients treated with an integrative therapy package (including HT) during chemotherapy treatment and conclude that the intervention warrants further investigation. Gronowicz et al. [8] utilized Therapeutic Touch (bioenergy) in a breast cancer mouse model with 4T1 modified 66c14 (highly metastatic to lymph nodes breast cancer cell line) and was able to demonstrate significant downregulation of specific lymphocytes, macrophages, and serum cytokines.

A review by Jackson et al. [25] concluded that there is clear support for the efficacy of energy healing (Healing Touch, Therapeutic Touch, and Reiki) in the treatment of pain and anxiety associated with cancer and cancer treatment. The effects of another energy therapy,* Qigong*, in the treatment of cancer have been extensively investigated in clinical studies in China. Chen and Yeung [26] concluded that although some of the studies were weakened by the absence of an adequate control, Qigong treatment appeared to be associated with significant clinical improvement and even spontaneous remission of late stage or metastasized cancer. These results are certainly clinically significant enough to warrant further investigation.

Agdal et al. [27] reviewed eight studies investigating the effects of energy healing on multiple cancer-related symptoms and state that, despite some promising results, definitive conclusions cannot be drawn due to serious methodological problems. Among the most egregious methodological violations are a lack of adequate blinding, self-selection of study participants (nonrandom sampling), small sample sizes, patient expectations (placebo effect), and inadequate controls. Multiple authors have called for better-controlled studies that adhere to existing standards for clinical trials [19, 27–29].

Scientific investigation of energy therapy is fraught with the potential for confounding due to placebo effects. As a result, many researchers have turned to* in vitro* and* in vivo* studies investigating the effects of energy healing on cancer cells cultured in a laboratory setting [25]. Positive results have been found in terms of inhibition of cancer cell growth and promotion of cell death and are not limited to particular laboratories, cell lines, practitioners, or types of intervention [25, 27, 28]. Yan et al. [30] treated human pancreatic cancer cells and healthy fibroblasts with external Qigong. One of the risks of chemotherapy is that healthy cells are damaged along with cancer cells but Yan et al. found that Qigong inhibited tumor growth pathways and did not exhibit cytotoxic effects on healthy fibroblasts. The ability to differentially affect cancerous and noncancerous cells could be key to developing safer treatment options.

Yu et al. [31] demonstrated the inhibition of growth of androgen independent human prostate cancer cells treated with energy emitted by a Zen master when compared to untreated cells. Significant differences between the groups were observed within 48 hours and increased over time. The slowed growth was not due to cytotoxicity or cell death. Androgen independent prostate cancer tends to be highly resistant to conventional treatment options [32], rendering these results particularly encouraging.

Abe et al. [33] utilized Johrei (Japanese energy healing) on various types of human cancer cells in culture and were able to show loss of viability for those cultured human cancer cells in the Johrei groups. The loss of viability was mainly due to increased cell death and decreased proliferation of cells for those treated groups.

The effects of Therapeutic Touch on the DNA synthesis, differentiation, and mineralization of normal human bone cells and bone cancer cells were investigated by Jhaveri et al. [34]. Two weeks of Therapeutic Touch treatment of* in vitro* cells resulted in increased DNA synthesis, differentiation, and mineralization of the healthy cells compared to untreated cells. Cancerous cells, on the other hand, showed decreased differentiation and mineralization. Again, this differential response indicates that energy therapy may offer a uniquely safe and effective cancer treatment option.

*In vivo* studies of the effects of energy healing on cancer progression generally involve experimental rodents that have been injected with cancerous cell lines known to produce tumors within a predictable time frame. Efficacy is evaluated in terms of tumor growth and/or survival time. These animal models allow researchers to observe the course of cancer in the whole organism over time. Chen et al. [35] investigated the effects of external Qigong treatment on growth of lymphatic tumors in mice. In their first study where mice received the treatment for 10 minutes every other day (a total of 4 treatments) and were sacrificed on day 9 or 11, tumor growth was slowed in the Qigong treatment compared to a sham treatment and control. In the second study where mice received the treatment for 10 minutes every other day (a total of 5 treatments) and were sacrificed on day 10 or 13, the effect was smaller and not statistically significant, despite an increase in the number of treatments. For both studies, lymph nodes were smaller for the treatment mice than for the control groups. Tumor growth continued when treatment was stopped, suggesting that the best results may be obtained with continued energy therapy.

Bengston and Krinsley [36] observed an interesting pattern of results in mice injected with breast cancer cells. This procedure reliably results in the development of a large tumor, which kills the host after 14–27 days. Mice in the experimental group were treated with an energy healing technique described by the authors as a “laying on of hands.” Tumors in treated mice ulcerated, failed to develop infection, and were absorbed back into the body, and the mice lived a normal life span. Tumor progression in untreated mice followed the predicted pattern and the animals died within the anticipated time frame. These results were achieved in multiple settings, with trained and untrained (as well as skeptical) healers. Some of the mice in the experimental condition were subsequently reinjected with the cancer cells and failed to develop the characteristic tumor, suggesting the development of immunity.

More recently, Gronowicz et al. [8] were able to show that Therapeutic Touch significantly reduced mouse breast cancer metastasis in a study of Balb/C female mice injected with the 4T1 (66c14) mouse mammary carcinoma. In the first pilot study, mice were treated for 10 minutes with Therapeutic Touch twice a week for 2 weeks, and in the second pilot mice were treated with the same intervention twice a week for a period of 4 weeks. In both studies, the rate of metastasis was significantly decreased.

Further* in vivo* evidence for the efficacy of energy therapy in the treatment of cancer comes from Xue-Feng et al. [37]. In this study mice receiving chemotherapy were treated with Qigong for 30 minutes twice a week for two weeks. Mice were euthanized two weeks after the completion of all treatments. The authors found that external Qigong, especially when used in combination with more traditional chemotherapy treatment, significantly inhibited tumor growth in mice. In addition, this combined treatment helped to restore compromised immunologic functions.

Some theorists conceptualize magnetic field therapy as similar to energy healing because both techniques utilize energy to treat disease and increase the body's self-healing capacity. The primary difference between these modalities is that energy healing is mediated by a caring practitioner. Tatarov et al. [38] investigated the effects of magnetic fields on mice injected with breast cancer cells. This study utilized a unique imaging technique whereby the cancer cells were labeled with firefly luciferase, allowing real-time observation of tumor growth. Mice in the experimental condition were exposed to magnetic fields for 60, 180, or 360 minutes daily for up to four weeks. Mice in the 360-minute group showed a 44-fold increase in tumor growth over the four weeks compared to 200–900-fold increase in the other groups. The tumors in the 360-minute group also showed significantly more necrosis than those in untreated controls. Moreover, mice not injected with cancer cells but exposed to the magnetic fields did not show any abnormalities in the lung, liver, or mammary gland tissue, indicating that this treatment may be safer than some more traditional interventions.

Although results in the literature are mixed, both* in vitro* and* in vivo* studies consistently suggest that there may be a place for energy therapy in the treatment of a range of cancers and cancer-related symptoms. However, this area of study is plagued by difficulties with replication; subsequent experiments conducted within the same laboratory do not always yield comparable results [26, 28]. The current study explores the effects of HT on tumor growth and metastasis in mice injected with breast cancer cells. The aims of the study were (1) to determine whether mice treated with HT demonstrated slowed tumor growth when compared to untreated controls, (2) to determine whether mice treated with HT demonstrated less frequent presence of metastatic cells compared to untreated controls, and (3) to determine whether positive control groups in different rooms had different outcomes (tumor size and metastasis).

## 2. Methods and Materials

### 2.1. Design

**Figure 1:**
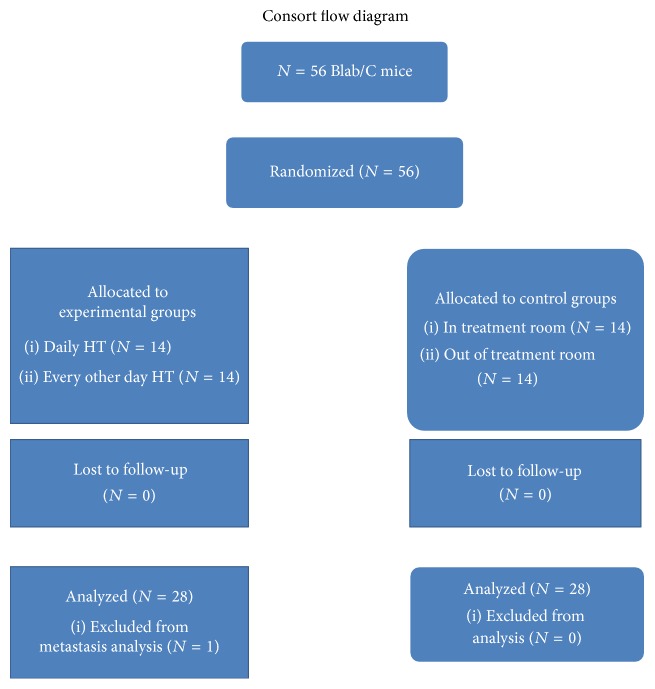
Treatment protocol.

This study used a randomized, four-group design. Two groups of mice that were exposed to the intervention (daily HT and every other day HT) were compared to each other and to two groups of positive control mice (one group located in the same room as the mice receiving HT and one group located in a different part of the building). Data (weight and tumor size) were collected at baseline and every three days until termination of study. At the end of the study, metastasis and final tumor sizes were measured for each mouse. These measurements were conducted by trained research assistants. This research was conducted under a protocol approved by the University of Nevada, Reno, Institutional Animal Care and Use Committee (IACUC) as described in Figure 1.

### 2.2. Sample

Fifty-six six- to eight-week-old male BALB/c mice (15–25 g) were obtained from Charles River Laboratories in California, USA. The mice were housed in a ventilated barrier rack in a temperature controlled facility on a 12-hour photoperiod. The mice were given food and water* ad libitum*. Mice were randomly assigned to 4 different cage sets (14 mice per cage set, 7 mice per cage) when they arrived to the Animal Resource Center (ARC) at the University of Nevada, Reno, and remained in those groups for the duration of the study.

### 2.3. Procedure

Five days after arrival, allowing time for acclimatization to the ARC and their group, each study mouse was injected with 0.1 mL of 4T1 murine mammary breast cancer cells subcutaneously in the lower right mammary gland (100,000 cells/dose). Five days after injection, allowing time for tumor establishment, the ten-day HT intervention for each group began. Two positive control groups (mice injected with cancer cells but no HT) were used in this study. One of these positive control groups remained in the same room as the intervention groups. A second positive control group was placed in a separate room, well away from the intervention room, in an effort to compare tumor size and metastasis outcomes. Tumor measurements and weights were gathered on five separate occasions for all mice (intervention and control) at the same time, by the same researcher to avoid bias and/or error. The certified HT practitioner was not blinded to the study, because of her need to deliver the intervention to each of the two groups. The HT practitioner was not provided with any measurement data taken during the study.

### 2.4. Protocol

Mice in the daily intervention group received ten consecutive, ten-minute HT treatments. The length of intervention was chosen after reviewing comparable studies. Mice in the every other day intervention group received five, ten-minute HT treatments. Length of treatment time was established after a review of the literature. Control mice did not receive any HT intervention. For both sets of control mice, employees of the ARC cleaned the cages and replaced food and water on the same dates and times. Apart from this interaction for maintenance, mice in the second control (in a different part of the building) had no contact with humans. No effort was made to tailor the HT treatments to any one mouse in the group.

Cages were clearly marked indicating each intervention group (daily or every other day), and prior to the beginning of the study the certified HT practitioner (who had been practicing for 10 years) was familiarized with the housing unit and clearly understood the method used for marking cages. Each cage set (daily and every other day) was placed side by side in the rack, separated from each other and the control group by two rows of empty cages. The cages were placed diagonally on the rack in an effort to mitigate any cross contamination or spillage of intervention between groups. The mice were housed seven to a cage, and each group (daily and every other day and positive control) contained 14 mice (as suggested by a power analysis).

**Figure 2:**
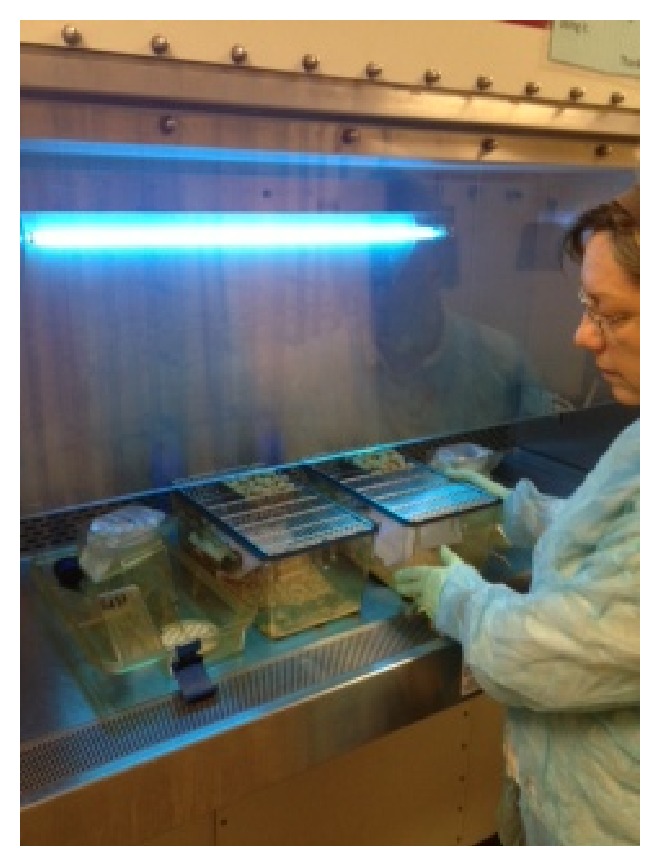
Healing Touch practitioner.

At the same time every day, the HT practitioner would gown, enter the room where the treatment mice were kept, glove, move the appropriate cages from the rack, and place them side by side under the hood. The plastic cover of the cage, along with the water that is situated inside the cage, was moved to the side of the hood leaving the metal slotted cover over each cage intact providing access to the mice for the practitioner (Figure 2). At no time did the HT practitioner have any physical contact with the mice.

The HT practitioner prepared for the session by centering and aligning herself, attuning to the mice and assessing their energy fields, and setting the following intentions: (1) energy will focus on this cage only, meaning the treatment intervention would not expand to other mice on the rack in the room, (2) cellular vibration level will be increased for each mouse to dissolve the tumor(s), (3) the blood supply will be diminished to the tumor cells, and (4) no metastasis will occur.

To accomplish those intentions, the bioenergy practitioner used a “hand scan” over the cages to determine levels of energy or auric field for each group. The practitioner would then “hold the field” to intensify energy to the mice. “Pain drain” was used to drain away irregularity from a specific or general area of the mouse bodies. “Hands in motion” would then be used to soothe and calm the field, and “hands still” was used to energize the spleen and adrenals. At the end of each HT intervention, after replacing the water and plastic cover over each of the cages, the practitioner would acknowledge the contribution of the mice, remove herself from the mouse energy field, place the mice back in their spot on the rack, remove her protective clothing, and leave the room (Figure 2). The mice were not disturbed until the next treatment time, apart from having their water and food checked by ARC staff.

On day ten of the intervention arm of the study all mice were euthanized following the IACUC protocol, tumors were measured (length × width) in mm^2^, and tissue samples from the tumor, liver, lung, and spleen were sent to the lab for processing. These organs were easily assessable and are known to be sites for metastasis [39].

### 2.5. Statistical Methods

For both the tumor size and the proportions of mice with metastasis across the four groups (treatment daily and every other day and control groups in the same and separate locations), an initial test for some difference in the groups was performed. Tumor sizes were analyzed using one-way ANOVA and model assumptions were reasonably met for this analysis. The original measurements were on an ordinal scale from 0 (none) to 4 (severe) metastases. After preliminary analysis, the responses were simplified to presence/absence of metastasis for each mouse and groups were compared using chi-square tests. The results were similar when analyzing the ordinal responses, so results are not presented here. All analyses were conducted using the statistical software R [40] with the plots made using gplots [41]. A power analysis was conducted prior to commencing the study for the tumor sizes with the suggested number of mice per group being typical for these types of studies.

## 3. Results

Balb/C mice were injected with 4T1 breast cancer cells to study the effects of HT. While no results were statistically significant, mice treated with HT had smaller tumors on average and lower proportions of metastasis. Mice treated with HT every other day had tumor sizes that were smaller on average, while mice housed away from the intervention group had the largest average tumor sizes. Metastasis was lowest for mice who received HT daily followed closely by those mice who received HT every other day. There was a very slight increase in metastasis for those positive control mice who were housed with the intervention groups but they were not statistically different from those positive control mice housed away from the intervention group.

**Figure 3:**
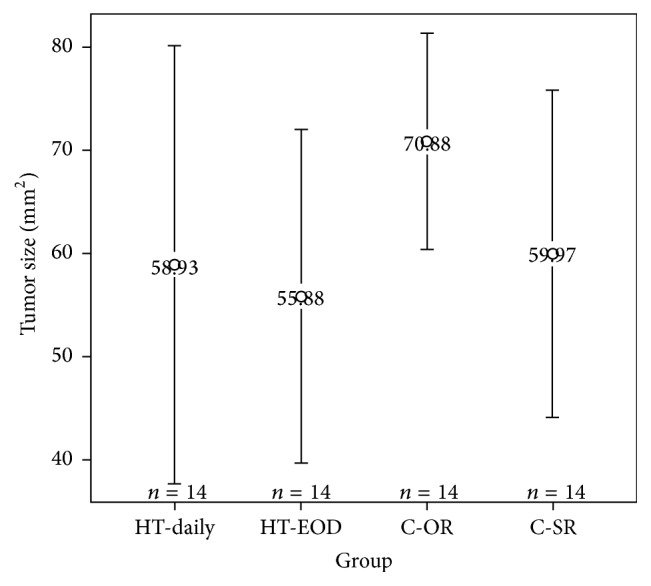
Plot of mean tumor area in mm^2^ by treatment group with 95% confidence intervals. Average tumor size is largest in the control group of mice housed in a separate room (C-OR) and is similar in the daily healing touch (HT-daily) group, the every other day healing touch (HT-EOD) group, and the control group housed in the same room as mice receiving healing teaching (C-SR). Average tumor sizes are not statistically significantly different.

The tumor size analysis did not demonstrate evidence of difference in the means between the four groups (*F*(3,52) = 0.75, *p* value = 0.53). The average tumor size was smallest in the HT-every other day group and largest in the Ctrl—other room group as seen in Table 1 and Figure 3. These differences are not statistically significantly different but may suggest a dose effect of HT on reduced tumor growth and that the every other day treatment dosage may be slightly better than daily treatment in limiting tumor growth.

**Figure 4:**
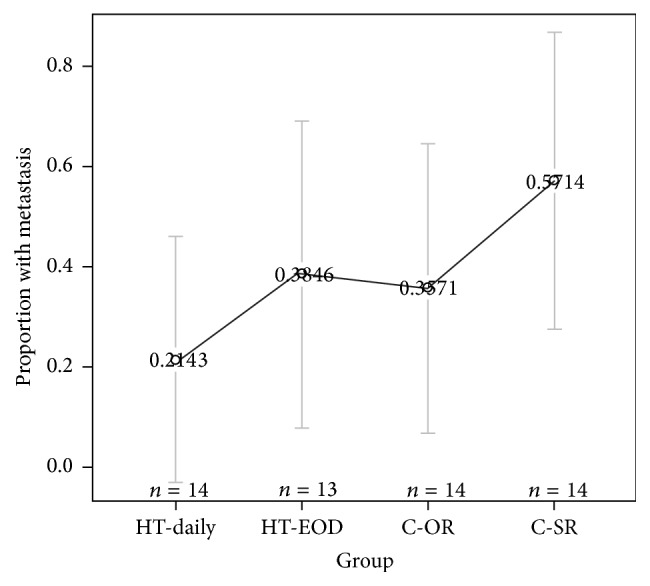
Plot of estimated proportions of each group with metastasis with 95% confidence intervals. Proportion of mice with metastasis is highest in the control mice housed in the same room (C-SR) as the mice receiving Healing Touch. The proportion of mice with metastasis is lower and similar in mice receiving Healing Touch every other day (HT-EOD) and mice not receiving Healing Touch and housed in a separate room (C-OR). Mice receiving Healing Touch daily have the lowest proportion of metastasis. The differences in proportions are not statistically significantly different.

The proportion of mice with metastasis does not differ statistically across treatment groups (chi-square(3) = 3.902, *p* = 0.272) with detailed results for the groups in Table 2 and Figure 4. Of note the lowest rate of metastasis occurred in the HT-daily group and the highest proportion was observed in the control group of mice housed in the same room as the mice receiving Healing Touch therapy.

The proportion of mice with metastasis was also analyzed by organ (liver, lung, and spleen) to evaluate if presence of metastasis differs across treatment groups for each organ. Metastasis rates are noticeably higher in the liver and lungs than the spleen for all four groups (Table 3). The proportion of mice with metastasis in the liver, lung, and spleen did not differ statistically (liver: chi-square(3) = 2.507, *p* = 0.474; lungs: chi-square(3) = 3.804, *p* = 0.283; spleen: chi-square(3) = 0.595, *p* = 0.898) with detailed results in Table 3. Metastasis rates in the intervention groups are higher in mice treated every other day than those treated every day. Unlike the results in Table 1, where the differences are greatest between those mice housed together and those mice housed in a separate room, the positive control mice housed with the intervention mice had the highest overall rate of metastasis (Table 2). For the control groups, metastasis rates are higher in mice located in the same room as mice in the intervention group than those housed in a separate room. These differences were not large enough given the sample of *n* = 14 mice per group to detect differences but do suggest the potential for HT-every day to have a dose effect on metastasis rates relative to the control group.

## 4. Conclusions

Bioenergy (Healing Touch) did not significantly impact tumor growth or metastasis in this study, but the results show a trend towards a decrease in tumor size for the treatment mice and a decrease in metastasis for the mice that were separate from the control groups. The results do suggest the potential for HT to be having a small positive effect on tumor size and metastasis rates but it is not clear which dosage level is better on each aspect of tumor development and the statistical support for the observed differences is very weak. While this study fails to identify significant differences, it adds to the information related to the impacts of HT and can provide useful information for designing future studies. Earlier studies looking at biofield energy and metastasis provided the intervention for a longer period of time, perhaps shedding light on a potential dose effect [8]. The failure to detect significant differences could be due to a chance result masking differences that the time frame was not long enough to detect differences or for the dosage of the treatments to have a noticeable impact.

One of these studies aims was to determine transference of biofield energy to mice in the same room and the results would indicate that, for tumor size, perhaps human contact had an impact on psychosocial stress factors and the immune system impacted tumor growth. However this cannot be transferred to metastasis.

As noted in the literature review, animal studies are very divergent. The same can be said for human studies in terms of duration of treatment and treatment outcomes. Some studies, such as this one, were conducted during a 10-day treatment period. Other studies last from 9 [25] to 24 [36], 30 [36, 37], or more days. While reported results are encouraging, none with the exception of the Bengston and Krinsley [36] studies are conclusive in regard to a cancer cure. As more people with cancer seek out complementary and integrative therapies, it is important that evidence is available to guide them in their therapy selections. Researchers, encouraged by these initial findings, can now move in the direction of setting up research parameters to more fully explore levels of dosage, frequency, or duration.

## Figures and Tables

**Table 1:** Summary statistics of tumor size by treatment group. No statistically significant differences in mean tumor size across the four treatment groups were found.

Treatment group	Mean (mm^2^)	*n*
HT-every day	58.93 ± 9.78	14
HT-every other day	55.88 ± 7.44	14
Control-same room (C-SR)	59.97 ± 7.31	14
Control-other room (C-OR)	70.88 ± 4.85	14

**Table 2:** Proportion of mice with metastasis by treatment group. The proportion of mice with metastasis does not differ statistically significantly across the four treatment groups.

Treatment group	Proportion	*n*
HT-every day	0.2143	14
HT-every other day	0.3846	13
Control-same room (C-SR)	0.5714	14
Control-other room (C-OR)	0.3571	14

**Table 3:** Proportion of mice with metastasis by organ and treatment group. For each organ examined the differences in the proportion of mice with metastasis are not statistically significantly different.

Treatment group	Liver	*n*	Lungs	*n*	Spleen	*n*
HT-every day	0.1429	14	0.0769	13	0.0714	14
HT-every other day	0.1538	13	0.1538	13	0.1538	13
Control-same room	0.3571	14	0.3571	14	0.1429	14
Control-other room	0.2857	14	0.1426	14	0.1426	14
